# Discrepancy between self-assessed hearing status and measured audiometric evaluation

**DOI:** 10.1371/journal.pone.0182718

**Published:** 2017-08-08

**Authors:** So Young Kim, Hyung-Jong Kim, Min-Su Kim, Bumjung Park, Jin-Hwan Kim, Hyo Geun Choi

**Affiliations:** 1 Department of Otorhinolaryngology-Head & Neck Surgery, CHA Bundang Medical Center, CHA University, Seongnam, Korea; 2 Department of Otorhinolaryngology-Head & Neck Surgery, Hallym University Sacred Heart Hospital, Anyang, Korea; 3 Department of Otorhinolaryngology-Head & Neck Surgery, Korea University Ansan Hospital, Ansan, Korea; 4 Department of Otorhinolaryngology-Head & Neck Surgery, Hallym University College of Medicine, Seoul, Korea; Ear Science Institute Australia, AUSTRALIA

## Abstract

**Objective:**

The purpose of this study was to examine the difference between self-reported hearing status and hearing impairment assessed using conventional audiometry. The associated factors were examined when a concordance between self-reported hearing and audiometric measures was lacking.

**Methods:**

In total, 19,642 individuals ≥20 years of age who participated in the Korea National Health and Nutrition Examination Surveys conducted from 2009 through 2012 were enrolled. Pure-tone hearing threshold audiometry (PTA) was measured and classified into three levels: <25 dB (normal hearing); ≥25 dB <40 dB (mild hearing impairment); and ≥40 dB (moderate-to-severe hearing impairment). The self-reported hearing loss was categorized into 3 categories. The participants were categorized into three groups: the concordance (matched between self-reported hearing loss and audiometric PTA), overestimation (higher self-reported hearing loss compared to audiometric PTA), and underestimation groups (lower self-reported hearing loss compared to audiometric PTA). The associations of age, sex, education level, stress level, anxiety/depression, tympanic membrane (TM) status, hearing aid use, and tinnitus with the discrepancy between the hearing self-reported hearing loss and audiometric pure tone threshold results were analyzed using multinomial logistic regression analysis with complex sampling.

**Results:**

Overall, 80.1%, 7.1%, and 12.8% of the participants were assigned to the concordance, overestimation, and underestimation groups, respectively. Older age (adjusted odds ratios [AORs] = 1.28 [95% confidence interval = 1.19–1.37] and 2.80 [2.62–2.99] for the overestimation and the underestimation groups, respectively), abnormal TM (2.17 [1.46–3.23] and 1.59 [1.17–2.15]), and tinnitus (2.44 [2.10–2.83] and 1.61 [1.38–1.87]) were positively correlated with both the overestimation and underestimation groups. Compared with specialized workers, service workers, manual workers, and the unemployed were more likely to be in the overestimation group (1.48 [1.11–1.98], 1.39 [1.04–1.86], and 1.50 [1.18–1.90], respectively), and service workers were more likely to be in the underestimation group (AOR = 1.42 [1.01–1.99]). Higher education level (0.77 [0.59–1.01] and 0.43 [0.33–0.57]) and hearing aid use (0.36 [0.17–0.77] and 0.23 [0.13–0.43]) were negatively associated with being in the underestimation group (0.43 [0.37–0.50]). Compared with males, females were less likely to be assigned to the underestimation group (0.43 [0.37–0.50]). Stress (1.98 [1.32–2.98]) and anxiety/depression (1.30 [1.06–1.59]) were associated with overestimation group.

**Conclusion:**

Older age, lower education level, occupation, abnormal TM, non-hearing aid use, and tinnitus were related to both overestimation and underestimation groups. Male gender was related to underestimation, and stress and anxiety/depression were correlated with overestimation group. An understanding of these factors associated with the self-reported hearing loss will be instrumental to identifying and managing hearing-impaired individuals.

## Introduction

The estimated prevalence of hearing loss is as high as approximately 16.1% and increases with age [1]. It has been reported that two-thirds of individuals over the age of 70 years in the United States have a hearing impairment [2]. Similarly, it has been estimated that 84.9% of Koreans aged 80 years or older exhibit bilateral hearing impairment, while approximately 6% of the young individuals (younger than 50 years of age) presents bilateral hearing impairment [3]. Due to the high prevalence of hearing impairment, the proper interpretation of self-reported hearing loss is crucial for predicting audiometric hearing loss.

Several previous studies have demonstrated that only 43–71% of individuals show concordance between their self-reported hearing loss and audiometric pure-tone audiometry (PTA) results [4,5]. Some individuals perceive their hearing impairments as greater than their audiometric PTA thresholds, and their complaints regarding their hearing abilities are not proportional to their audiometric PTA results [6]. In contrast, other individuals showed low self-reported hearing loss compared to their audiometric PTA results [6]. The resulting missed diagnoses of hearing difficulty delay hearing rehabilitation with hearing aids, implantable devices, counseling, or training [7]. Therefore, it is necessary for these individuals to be aware of the factors related to low perceive relative to their audiometric PTA threshold so that they can detect and manage their hearing impairment. Similarly, individuals who report high perceive with their hearing difficulty relative to their audiometric PTA threshold (and who do not exhibit such difficulty during audiometric PTA testing) also need to be managed by clinicians. Information on the factors which may be related to the discrepancy between self-reported hearing loss and audiometric hearing loss may assist clinicians in the treatment of these individuals.

Several factors are associated with biases in self-reported hearing loss. It has been reported that age, sex, ethnicity, education level, and the degree of measured hearing impairment are associated with the self-reported hearing loss [6,8]. The older adults are more likely to have a high discrepancy between audiometry and self-report [9,10]. In this context, factors related to auditory problems, such as stress, anxiety, occupation, middle ear infection (which can compromise the tympanic membrane (TM) integrity), and tinnitus might be considered when evaluating self-reported hearing difficulties [11–13]. Furthermore, factors related to the respondent’s personality—particularly, his/her stress coping style and neuroticism—may be associated with his/her self-reported hearing loss [14].

The present study aimed to evaluate the factors associated with discrepancies between self-reported hearing loss and audiometric PTA results. There are several studies examining the factors associated with the discrepancy between self-reported hearing loss and audiometric findings, however most of these studies have been confined to the elderly population, ages 50 and older[6,15,16].Few studies have focused on the otologic and psychosocial factors that might influence the perception of hearing impairment in large, representative populations with a wide range of age groups. Moreover, previous studies have evaluated hearing using a dichotomized classification [4,15]. A recent study reported that the 80% of older adults with self-reported hearing loss demonstrated at least mild hearing impairment during audiometric PTA measurements [17]. In the present investigation, the degree of hearing impairment was classified into three groups. To the best of our knowledge, this study is novel in its multinomial logistic regression analysis of the relationships among various factors (including otologic factors) and patterns (i.e., high perceived hearing difficulty and low perceived hearing difficulty) related to perceived hearing difficulty.

## Materials and methods

### Study population and data collection

The present study was approved by the institutional review board of the Korea Centers for Disease Control and Prevention (2009-01CON-03-2C, 2010-02CON-21-C, 2011-02CON-06-C, 2012-01EXP-01-2C). Written informed consent was obtained from each participant prior to the survey.

This investigation was a cross-sectional study that utilized data from the Korea National Health and Nutrition Examination Survey (KNHANES), which included participants who were representative of the South Korean population. The survey included a health interview, a nutritional survey, and physical examinations. The applied statistical methods were based on the sampling design and utilized weighted values. The KNHANES data collected by the Centers for Disease Control and Prevention of Korea from 2009 to 2012 were analyzed. Each year, 192 districts were selected by a panel, and 20 households in each of these districts were further identified to allow sampling that reflected the entire Korean population. The surveys evaluated data from the civilian, non-institutionalized South Korean population using a stratified, multistage, clustered sampling method based on the national census data. The sample was weighted by statisticians who performed post-stratification and accounted for the non-response rates and extreme values.

Among the 36,067 total participants, we excluded the following: participants younger than 20 years old (8,875 participants), because children and adolescents were not surveyed regarding their social economic status; participants who refused or failed a PTA test (7,097 participants); and participants who reported incomplete data regarding educational level, occupation, stress level, anxiety/depression, TM, hearing aid use, and tinnitus history (453 participants). Ultimately, 19,642 participants (8,393 males and 11,249 females) were included in this study. The survey participants ranged in age from 20 to 97 years. The mean age of the overall population was 50.4 ± 16.2 years. All the enrolled individuals had sufficient cognitive function to complete the questionnaire, the physical examination, and PTA.

### Measures

In this study, age, sex, education, occupation, stress level, anxiety/depression, TM status, hearing aid use (including implantable devices), and tinnitus were used as independent variables. The discrepancy between self-reported hearing loss and the audiometric PTA results (three groups: concordance, overestimation, and underestimation) was used as the dependent variable.

The patients were divided into the following three groups based on education level: middle school or less (low), high school or junior college (middle), and college or graduate school (high). Ten types of occupations based on the Korean standard occupation classification were used [18]. The ten types of occupation were manager; expert, specialist; clerk; service worker; seller; farmer, fisher; technician, mechanic, production worker, engineer; laborer; soldier. An unemployed group was added to create 11 groups. The classifications were re-categorized into four groups according to their physical activity levels [19,20], as follows: managers, experts, and specialists (Specialized workers); clerks, service workers, salespeople, technicians, mechanics, production workers, and engineers (Service workers); and farmers, fishers, laborers, and soldiers (Manual workers). The unemployed participants formed a fourth group (Unemployed).

#### Questionnaire

The participants were asked about their stress level (“How much do you feel stress in ordinary life?”) and presented with 4 possible answers: “I feel little stress”, “I feel some stress”, “I feel much stress”, and “I feel very stress”. The participants were asked questions about their anxiety/depression level using a translated EQ-5D-3 L question [21]. They selected among 3 responses: “I am not anxious or depressed”, “I am moderately anxious or depressed”, and “I am extremely anxious or depressed”. The participants were asked whether they had heard any ringing, buzzing, roaring, or hissing sounds in the absence of an external acoustic source within the past year. The response options were “Yes”, “No”, and “I cannot remember”. The 29 participants who answered “I cannot remember” were grouped with the participants who answered “No”. In the present study, each participant was examined by trained otorhinolaryngology residents using a 4-mm, 0°-angled rigid endoscope. TM images were recorded using a charge-coupled-device (CCD) camera and reviewed by otorhinolaryngology specialists. The TM findings were used to categorized the right and left TMs into the following three groups: normal, unilaterally abnormal, and bilaterally abnormal. An abnormal TM was defined as a TM with perforation, cholesteatoma, middle ear effusion, ventilation tube, or retraction [22].

#### Audiometric evaluation

After the completion of the questionnaire and physical examination, the audiometric PTA were measured in a soundproofing booth using an audiometer (SA 203, Entomed, Sweden), and included frequencies of 500, 1000, 2000, 3000, 4000, and 6000 Hz in both ears in accordance with the American National Standards Institute (ANSI) standard. The hearing threshold was defined as the mean hearing threshold of the better ear on PTA tests at 500, 1000, 2000, and 4000 Hz. Hearing loss in this study was defined as follows: <25 dB (normal hearing), ≥25 dB and <40 dB (mild hearing loss), and ≥40 dB (moderate-to-severe hearing loss).

#### Self-reported hearing loss

The participants were instructed to “Please select one answer below that most accurately expresses your hearing status without hearing aids”. The possible answers were “I feel no difficulty”, “I feel some difficulty”, “I feel much difficulty”, and “I cannot hear”. Next, we grouped the participants who responded “I feel much difficulty” and “I cannot hear” together into one group, which is subsequently referred to as the “much difficulty” group.

Next, we divided the participants into three groups according to the difference between their perception of their hearing impairment and their audiometric PTA results (S1 Table). The concordance group comprised those whose self-reported hearing loss was similar to their audiometric PTA results and who reported no difficulty hearing at <25 dB HL, some difficulty at ≥25 dB HL and <40 dB HL, and much difficulty at ≥40 dB HL hearing thresholds. The overestimation group comprised those who perceived greater self-reported hearing loss relative to their audiometric PTA results and reported some difficulty at <25 dB HL and much difficulty at <40 dB HL hearing thresholds. The underestimation group comprised those who perceived less self-reported hearing loss than their audiometric PTA level suggested and reported no difficulty at ≥25 dB HL and some difficulty at ≥40 dB HL hearing thresholds. In this study, ‘overestimation group’ refers to the participants who reported self-reported hearing loss that was high ‘compared with their audiometric PTA thresholds’. This group could be expressed as having either a ‘good audiometric PTA’ or a ‘low audiometric PTA threshold’ relative their self-reported hearing loss. For readability, we used the audiometric PTA thresholds as the reference and chose to use the term ‘overestimation group’.

### Statistical analysis

The differences in general characteristics according to the discrepancy between self-reported hearing loss and measured audiometric evaluation were calculated using analysis of variance (ANOVA) tests for age and hearing threshold and chi-square tests for education level, occupation, stress level, anxiety/depression, TM status and tinnitus.

The associations of the examined factors (independent variables) with the discrepancy between self-reported hearing loss and audiometric PTA (i.e., concordance, high perceived and low perceived groups) were analyzed using multinomial logistic regression with complex sampling. Multinomial logistic regression is an extension of logistic regression for use when a nominal outcome has more than two unordered categories/levels. In this analysis, concordance group was set as the reference. Two-tailed analyses were conducted, and P values below 0.05 were considered significant. The adjusted odds ratios (AORs) and 95% confidence intervals (CIs) were calculated. All the results are presented as weighted values. The results were statistically analyzed using SPSS version 21.0 (IBM, Armonk, NY, USA).

## Results

In total, the 19,642 participants exhibited various degrees of hearing impairment (including no hearing impairment); specifically, 81.20% (15,949), 12.92% (2,537), and 5.89% (1,156) of the participants exhibited mean hearing thresholds <25 dB HL, ≥25 dB HL and <40 dB HL, and ≥40 dB HL, respectively. Overall, 80.1% (15,742), 7.1% (1,385), and 12.8% (2,515) of the participants were in the concordance, overestimation, and underestimation groups, respectively. Regarding hearing levels, the concordance decreased with increasing hearing thresholds, and 92.2% and 7.7% of the normal-hearing participants were in the concordance and overestimation groups, respectively. Among the participants with mild hearing impairment based on audiometric PTA, 28.1%, 5.8%, and 66.1% were in the concordance, overestimation, and underestimation groups, respectively. Of the participants with moderate-to-severe hearing impairment, 27.4% and 72.5% were in the concordance and underestimation groups, respectively (Table 1).

**Table 1:** Hearing perception according to self-reported hearing loss and objective hearing difficulty.

Self-reported hearing loss	Audiometric evaluation of hearing		
	<25 dB	≥25 dB<40 dB	≥40 dB
No difficulty	14,712* (92.2%)	1,676‡ (66.1%)	383‡ (33.1%)
A little difficulty	1,155† (7.2%)	713* (28.1%)	456‡ (39.4%)
Much difficulty	82† (0.5%)	148† (5.8%)	317* (27.4%)
Total	15,949 (100%)	2,537 (100%)	1,156 (100%)

* Concordance group: No self-reported hearing loss at a hearing threshold of<25 dB; a little self-reported hearing loss at ≥25 dB to <40 dB; much self-reported hearing loss at ≥40 dB.

†Overestimation group: A little self-reported hearing loss at <25 dB; much self-reported hearing loss at both <25 dB and ≥25 dB to <40 dB.

‡Underestimation group: No self-reported hearing loss at both ≥25 dB to <40 dB and ≥40 dB; a little self-reported hearing loss at ≥40 dB.

We investigated several characteristics that were potentially associated with the discrepancy between self-reported hearing loss and audiometric PTA. Age, sex, education level, occupation, stress level, anxiety/depression, TM abnormalities, and tinnitus were correlated with the discrepancy between self-reported hearing loss and audiometric PTA (Table 2). Males (15.8%) were more prone to underestimation of hearing loss than females (10.5%) (P < 0.001), while females (7.6%) were more likely than males (6.3%) to experience overestimation of hearing loss (P < 0.001). As the education level decreased, the discrepancy between self-reported hearing loss and audiometric PTA increased with respect to both overestimation and underestimation of hearing loss (P < 0.001). The proportions of concordance between self-reported hearing loss and audiometric PTA differed significantly according to occupational group (P < 0.001). Specialized workers comprised the largest proportion of the concordance group (P < 0.001). Individuals with high stress levels were more likely to present overestimation of hearing loss (P < 0.001). Individuals with higher levels of anxiety/depression showed discrepancies between self-reported hearing loss and audiometric PTA results (P < 0.001). TM abnormalities and tinnitus were associated with both overestimation and underestimation of hearing loss (both P values < 0.001; Table 2).

**Table 2:** General characteristics of the hearing estimation groups.

	Groups			P-value
	Concordance	Overestimation	Underestimation	
Participants (number, %)	15,742 (80.1)	1,385 (7.1)	2,515 (12.8)	
Age (years)	47.1	55.4	68.1	<0.001*
Hearing threshold (dB)	11.7	16.3	38.7	<0.001*
Sex (number, %)				<0.001†
Male	6,532 (77.8)	532 (6.3)	1,329 (15.8)	
Female	9,210 (81.9)	853 (7.6)	1,186 (10.5)	
Education (number, %)				<0.001†
Low	4,446 (64.1)	661 (9.5)	1,833 (26.4)	
Middle	6,883 (87.4)	492 (6.3)	496 (6.3)	
High	4,413 (91.3)	232 (4.8)	186 (3.9)	
Occupation (number, %)				<0.001†
Group 1	3,732 (92.5)	192 (4.8)	109 (2.7)	
Group 2	2,839 (89.5)	221 (6.7)	245 (7.4)	
Group 3	3,714 (74.1)	338 (7.9)	773 (18.0)	
Group 4	5,997 (74.8)	634 (7.9)	1,338 (17.3)	
Stress level (number, %)				<0.001†
None	2,147 (70.1)	175 (5.7)	739 (24.1)	
Some	9,258 (82.4)	742 (6.6)	1,238 (11.0)	
Moderate	3,639 (81.7)	381 (8.6)	432 (9.7)	
Severe	698 (78.3)	87 (9.8)	106 (11.9)	
Anxiety/depression (number, %)				<0.001†
No	14,122 (81.1)	1,124 (6.5)	2,162 (12.4)	
Moderate	1,512 (72.6)	243 (11.7)	328 (15.7)	
Extreme	108 (71.5)	18 (11.9)	25 (16.6)	
Tympanic membrane (number, %)				<0.001†
Normal, both	14,509 (82.0)	1,133 (6.4)	2,048 (11.6)	
Abnormal, unilateral	919 (63.7)	194 (13.5)	329 (22.8)	
Abnormal, bilateral	314 (61.6)	58 (11.4)	138 (27.1)	
Hearing aid use (number, %)				0.139
No	15,616 (80.2)	1,373 (7.1)	2,485 (12.8)	
Yes	126 (75.0)	12 (7.1)	30 (17.9)	
Tinnitus (number, %)				<0.001†
No	12,764 (83.3)	828 (5.4)	1,728 (11.3)	
Yes	2,978 (68.9)	557 (12.9)	787 (18.2)	

* ANOVA test, significance at P < 0.05

† Chi-square test, significance at P < 0.05

The multinomial logistic regression analysis revealed that older participants tended to show less concordance between their self-reported hearing loss and audiometric PTA results in age from 20 to 97 years. The discrepancy between self-reported hearing loss and audiometric PTA increased with age; for every 10 years of age, the rate of overestimation of hearing loss increased 1.28-fold (95% CI = 1.19–1.37), and the rate of underestimation of hearing loss increased 2.80-fold (95% CI = 2.62–2.99; P < 0.001). Females were less likely than males to have underestimation of hearing loss (AOR = 0.43, 95% CI = 0.37–0.50, P < 0.01). A high level of education was associated with the concordance of hearing impairment results, and the dose-response relationship was significant (the AORs [for both the high and low perceived hearing difficulty groups] indicated an education group ordering of High < Middle < Low; P < 0.001). Service workers, manual workers, and the unemployed were more likely than specialized workers to experience overestimation of hearing loss (AOR = 1.48, 1.39, and 1.50, respectively, P = 0.010). As stress levels increased, greater proportions of participants presented overestimation of hearing loss (AOR = 1.98, 95% CI = 1.32–2.98, P < 0.001). A moderate level of anxiety/depression was associated with overestimation of hearing loss (AOR = 1.30, 95% CI = 1.06–1.59, P = 0.003). The participants with abnormal TMs were more likely to have a discrepancy between their self-reported hearing loss and audiometric PTA results compared with participants with normal TMs (bilateral abnormal TM, AOR of high perceived hearing difficulty = 2.17 [95% CI = 1.46–3.23]; AOR of low perceived hearing difficulty = 1.59 [95% CI = 1.17–2.15]. P < 0.001). Tinnitus was related to the discrepancy between self-reported hearing loss and audiometric PTA results with respect to both overestimation and underestimation of hearing loss (AOR of overestimation of hearing loss = 2.44 [95% CI = 2.10–2.83]; AOR of underestimation of hearing loss = 1.61 [95% CI = 1.38–1.87]. P < 0.001; Table 3).

**Table 3:** Multinomial logistic regression analysis with complex sampling (reference = relevance group).

Related factors		Overestimation		Underestimation		
	%*	AOR	95% CI	AOR	95% CI	P-value
Age (10 years)		1.28	1.19–1.37	2.80	2.62–2.99	<0.001†
Sex						<0.001†
Male	49.7	1		1		
Female	50.3	0.86	0.73–1.01	0.43	0.37–0.50	
Education						<0.001†
Low	27.4	1		1		
Middle	45.1	0.85	0.70–1.04	0.66	0.56–0.79	
High	27.6	0.77	0.59–1.01	0.43	0.33–0.57	
Occupation						0.010†
Specialized worker	23.5	1		1		
Service worker	20.1	1.48	1.11–1.98	1.42	1.01–1.99	
Manual worker	20.8	1.39	1.04–1.86	1.14	0.85–1.53	
Unemployed	35.6	1.50	1.18–1.90	1.25	0.93–1.67	
Stress						0.001†
None	13.2	1		1		
Some	58.6	1.25	0.99–1.59	0.99	0.85–1.16	
Moderate	23.6	1.75	1.35–2.29	1.07	0.89–1.30	
Severe	4.6	1.98	1.32–2.98	1.33	0.93–1.88	
Anxiety/depression						0.003†
No	89.8	1		1		
Moderate	9.6	1.30	1.06–1.59	0.92	0.74–1.13	
Extreme	0.6	0.88	0.74–1.13	0.45	0.27–0.76	
Tympanic membrane						<0.001†
Normal, both	91.2	1		1		
Abnormal, unilateral	6.4	2.21	1.75–2.78	1.34	1.10–1.62	
Abnormal, bilateral	2.3	2.17	1.46–3.23	1.59	1.17–2.15	
Hearing aid use						<0.001†
No	99.4	1		1		
Yes	0.6	0.36	0.17–0.77	0.23	0.13–0.43	
Tinnitus						<0.001†
No	79.2	1		1		
Yes	20.8	2.44	2.10–2.83	1.61	1.38–1.87	

* Estimated rate, adjusted with weighted values

† Significance at P < 0.05

To evaluate the discrepancy between self-reported hearing loss and audiometric PTA results according to audiometric PTA thresholds (< 25 dB HL; ≥ 25 dB HL and < 40 dB HL; and ≥ 40 dB HL), a subgroup analysis was performed using logistic regression analysis. The results revealed that old age, occupation, and stress level were significantly associated with discrepancies only in the individuals with < 25 dB HL. TM abnormality and tinnitus were associated with a discrepancy between self-reported hearing loss and audiometric PTA in all the hearing threshold groups (S2 Table). Further analysis of self-reported hearing loss in the high-frequency hearing loss group using the average of 3000, 4000, and 6000 Hz demonstrated comparable results (S3 and S4 Tables).

## Discussion

### Principle findings of the present study

In our cohort, 80.1% belonged to the concordance group. This value is higher than those presented in previous studies, which reported rates of 43–71% [4,5]. The high rate of concordance in the present study may be explained by the fact that we included a population of young adults who generally perceive their hearing impairment more accurately than the older population, whereas most of the previous studies included only the elderly [5,23,24]. In addition, the ethnicity, prevalence of audiometric hearing loss, and socioeconomic status might attribute to the difference of the concordance rate among studies. A cross-sectional study based on the US population aged 20 to 69 years showed 41–65% of sensitivity of self-reported hearing loss [1]. A prospective study including young participants reported an 81% of sensitivity and 52% of specificity for television volume as a marker for hearing loss [25]. Moreover, we found that 7.1% of the participants presented overestimation of hearing loss and that 12.8% of participants exhibited underestimation of hearing loss. Several variables, including otologic factors, were found to influence concordance between self-reported hearing loss and audiometric PTA results. Each factor is discussed in detail in the following paragraphs.

### Demographics and self-reported hearing loss

Old age was related to overestimation and underestimation of hearing loss. The elderly tend to be more accepting of hearing difficulty than young individuals because their needs for communication in daily work and meetings are reduced [6]. Additionally, hearing impairment is often perceived as a normal aging process. As the prevalence of hearing loss increases with age, it may become more normal and thus less likely to be perceived as a problem that requires treatment. In some elderly individuals with auditory processing disorders, central auditory recognition problems in the absence of auditory perception disabilities can be subjectively perceived as hearing impairments [26]. These factors may be associated with the increased discordance between self-reported hearing loss and audiometric PTA test results with age. Moreover, diminishments in cognitive functions, such as spatial working memory and sustained visual attention, are more common in the elderly and have been reported to be related to self-reported hearing loss [27]. In this study, the male participants were more likely than the females to exhibit underestimation of hearing loss (AOR of females = 0.43, [95% CI = 0.37–0.50]). The gender norms or cultural notions of masculinity that affect male attitudes toward health and health service use might also promote the underestimation of hearing loss in males. In addition, a previous study suggested that the frequent exposure of males to noise may be related to insensitivities to hearing impairment [28]. Although there are some controversies related to the relevant study’s design, it has been shown that females express more hypochondriac concerns than males [29]. Moreover, because depression has been reported to be more prevalent in women, we presume that women are more likely than men to demonstrate overestimation of hearing loss [30]. However, female sex was not related to overestimation of hearing loss in this study (AOR = 0.86, [95% CI = 0.73–1.01]).

#### Emotional problems and self-reported hearing loss

Anxiety and depression were significantly related to overestimation of hearing loss in the present study. A series of studies has suggested the adverse effects of hearing impairments on depression and cognition, especially in the elderly [31,32]. In addition, strong correlations of depression and anxiety with self-reported hearing loss have been reported [33,34]. Similarly, some studies have suggested that personality traits strongly influence the self-reported hearing loss [14,35]. Increased sensitivity or anxiety in stressed individuals may sensitize them to hearing difficulties [36]. Accordingly, our results revealed a positive correlation between stress and overestimation of hearing loss. In contrast, adequate attention to hearing difficulty may be related to the concordance of hearing difficulty.

### Socioeconomic status and self-reported hearing loss

In the present study, specialized workers showed significantly least discrepancy between self-reported hearing loss and audiometric PTA results. On the other hands, unemployed showed significantly more overestimation of hearing loss. This result may be explained by the fact that individuals with hearing loss have more difficulty obtaining employment and are more frequently injured on the job [37]. A higher level of education was related to concordance between self-reported hearing loss and audiometric PTA results in the current study. These results are comparable to those of previous studies that demonstrated that age and education are related to the proper perception of hearing impairment [5,6,27]. Low education or income groups showed high prevalence of self-reported hearing loss as well as audiometric hearing loss [38]. More accessibility to medical contents and services of high education group could influence to the high concordance between self-reported hearing loss and audiometric hearing [38].

### Otologic findings and self-reported hearing loss

Tinnitus was related to both overestimation and underestimation of hearing loss. Like stressed individuals, those with tinnitus are vulnerable to anxiety regarding and hypersensitivity to hearing difficulty, which may result in their high discrepancy between measured audiometric hearing loss and self-reported hearing loss observed in this study. However, individuals with tinnitus may be confused about their hearing abilities because loud tinnitus can mask real sounds. Moreover, even tinnitus patients with a normal audiogram occasionally have hidden hearing impairment [39,40]. Consequently, tinnitus was found to be associated with both overestimation and underestimation of hearing loss.

The presence of a TM abnormality was associated with a discrepancy between self-reported hearing loss and audiometric PTA results. TM perforations, middle ear effusion, and other miscellaneous lesions associated with inflammatory conditions and can disturb sound conduction through the middle ear account for the majority of TM abnormalities. Such inflammatory reactions cause other complaints, such as middle ear discharge, otalgia, and facial nerve palsy. These complications may disturb the accurate perception of hearing impairment besides of the increased audiometric PTA air conduction threshold. Furthermore, inflammation and infection are temporary rather than permanent events. Middle ear pathologies can be gradually improved or aggravated, and such changes result in the improvement or aggravation of hearing impairments. This perturbation of hearing may impede the concordance of hearing ability. Furthermore, a histories of middle ear infection has been suggested to increase tinnitus independently from audiometric measures [13].

Hearing aid users exhibited significantly less overestimation (AOR = 0.36, 95% CI = 0.17–0.77, P < 0.001) and underestimation (AOR = 0.23, 95% CI = 0.13–0.43, P < 0.001) of hearing loss compared with non-hearing aid users. The overestimation of hearing loss of hearing aid users may be the result of the hearing aid users receiving information on hearing difficulty prior to the survey at previous hearing evaluations. In contrast, regarding the underestimation of hearing loss of hearing aid users, the audiometric PTA threshold may also influence the self-reported hearing loss and could be attributed to the relief of hearing difficulty as a result of wearing a hearing aid.

Our results showed that old age, occupation, and stress level were associated with the patterns of self-reported hearing loss in the individuals with<25 dB HL. However, because most of our study population (81.2%) had a hearing threshold of <25 dB HL, we could not find a discrete, statistically significant discrepancy between self-reported hearing loss and audiometric PTA in the ≥ 25 dB subgroup (S1 Table).

### Limitations of the present study

The current study has strengths relative to previous investigations in the comprehensive adjustments for various factors and the large representative population-based study design. We used the hearing thresholds from the better-hearing ears, which minimized the possibility of underestimation of hearing loss. The selection of the better ear was based on the classification of World Health Organization, which was adopted in many prior studies [6,15].

Despite these strengths, our study has limitations. There are conflicting opinions regarding the use of a reference for hearing thresholds [8,41,42]. We employed the reference value of 40 dB HL because it has been used in other studies [8,15]. Missing data, mainly resulting from individuals who refused to undergo an audiometric PTA test (7,097 participants), were excluded from the present analysis (S5 Table). Moreover, the individuals who had undergone a previous hearing evaluation may have perceived their hearing difficulties more accurately than the other participants. While this phenomenon could not be accounted for in this study, only approximately 1.8% (914,107/50,617,045) of individuals undergo audiometric PTA at medical check-ups in Korea [43]. Therefore, only a tiny portion of the enrolled participants may have undergone a previous hearing evaluation, and this effect would have little influence on the results of the present study. Because participants were not advised not to expose themselves to loud music or noise in the 24 hours prior to testing, temporary threshold shift could not excluded in the current audiometric PTA results [25,38]. Although otoscopic evaluations were performed to identify tympanic membrane abnormalities, tympanometry was not performed. Finally, questions for measuring stress level were not validated in this study.

Further studies to clarify the predictive values and patterns of self-reported hearing loss across a wide range of ages are warranted. Additional factors will be comprehensively investigated to determine their associations with the patterns of self-reported hearing loss. By helping to predict the patterns of self-reported hearing loss, these results will greatly aid the identification of hearing-impaired individuals. In addition, knowledge regarding self-reported hearing loss in association with patient characteristics and audiometric PTA thresholds will advance the management of hearing problems.

## Supporting information

S1 Table Classification of the discrepancies between self-reported hearing loss and audiometric PTA results. (DOCX)

S2 Table Subgroup analysis of self-perceived hearing difficulty using logistic regression analysis with complex sampling (reference = relevance group). (DOCX)

S3 Table Hearing perception according to self-reported and audiometric hearing loss at the high-frequency hearing threshold. (DOCX)

S4 Table Multinomial logistic regression analysis with complex sampling (reference = relevance group) at the high-frequency hearing threshold. (DOCX)

S5 Table Differences in the general characteristics of the participants who underwent pure tone audiometry and those who did not. (DOCX)
