# Supplementary material for: Discrepancy between self-assessed hearing status and measured audiometric evaluation
Source: PLoS One. 2017 Aug 8;12(8):e0182718. doi: 10.1371/journal.pone.0182718 (PMC5549722; doi:10.1371/journal.pone.0182718)
Supplement: S1 Table — (DOCX) [file pone.0182718.s001.docx]

| Self-reported hearing loss | Audiometric evaluation of hearing | | |
| --- | --- | --- | --- |
|  | <25 dB | ≥25 dB<40 dB | ≥40 dB |
| No difficulty | Concordance | Underestimation | Underestimation |
| A little difficulty | Overestimation | Concordance | Underestimation |
| Much difficulty | Overestimation | Overestimation | Concordance |

**S1 Table** Classification of the discrepancies between self-reported hearing loss and audiometric PTA results
