# Supplementary material for: Discrepancy between self-assessed hearing status and measured audiometric evaluation
Source: PLoS One. 2017 Aug 8;12(8):e0182718. doi: 10.1371/journal.pone.0182718 (PMC5549722; doi:10.1371/journal.pone.0182718)
Supplement: S2 Table — (DOCX) [file pone.0182718.s002.docx]

**S2 Table** Subgroup analysis of self-perceived hearing difficulty using logistic regression analysis with complex sampling (reference = concordance group)

| Subgroup | | <25 dB | | | ≥25 dB, <40 dB | | | | | ≥40 dB | | |
| --- | --- | --- | --- | --- | --- | --- | --- | --- | --- | --- | --- | --- |
| Related factors | | Overestimation | |  | Overestimation | | Underestimation | |  | Underestimation | |  |
|  | | AOR | 95% CI | P-value | AOR | 95% CI | AOR | 95% CI | P-value | AOR | 95% CI | P-value |
| Age (10 years) | | 1.33 | 1.24-1.44 | <0.001† | 0.85 | 0.65-1.12 | 0.99 | 0.88-1.12 | 0.516 | 0.90 | 0.72-1.13 | 0.356 |
| Sex | |  |  | 0.160 |  |  |  |  | 0.145 |  |  | 0.623 |
|  | Male | 1 |  |  | 1 |  | 1 |  |  | 1 |  |  |
|  | Female | 0.89 | 0.75-1.05 |  | 0.61 | 0.04-1.01 | 0.86 | 0.66-1.12 |  | 0.91 | 0.61-1.34 |  |
| Education | |  |  | 0.174 |  |  |  |  | 0.891 |  |  | 0.331 |
|  | Low | 1 |  |  | 1 |  | 1 |  |  | 1 |  |  |
|  | Middle | 0.84 | 0.69-1.04 |  | 0.96 | 9.53-1.76 | 1.01 | 0.73-1.40 |  | 0.74 | 0.44-1.23 |  |
|  | High | 0.80 | 0.60-1.05 |  | 0.62 | 0.24-1.59 | 0.93 | 0.59-1.45 |  | 0.63 | 0.27-1.48 |  |
| Occupation | |  |  | 0.009† |  |  |  |  | 0.215 |  |  | 0.884 |
|  | Specialized worker | 1 |  |  | 1 |  | 1 |  |  | 1 |  |  |
|  | Service worker | 1.52 | 1.13-2.04 |  | 0.68 | 0.15-3.19 | 1.34 | 0.7-2.38 |  | 0.65 | 0.15-2.81 |  |
|  | Manual worker | 1.40 | 1.04-1.88 |  | 1.10 | 0.25-4.76 | 1.03 | 0.59-1.78 |  | 0.60 | 0.16-2.27 |  |
|  | Unemployed | 1.50 | 1.18-1.90 |  | 2.00 | 0.45-8.23 | 1.26 | 0.72-2.20 |  | 0.59 | 0.16-2.20 |  |
| Stress | |  |  | 0.001† |  |  |  |  | 0.077 |  |  |  |
|  | None | 1 |  |  | 1 |  | 1 |  |  | 1 |  | 0.773 |
|  | Some | 1.29 | 1.00-1.66 |  | 0.90 | 0.46-1.74 | 0.83 | 0.62-1.11 |  | 1.11 | 0.73-1.67 |  |
|  | Moderate | 1.85 | 1.39-2.47 |  | 0.84 | 0.40-1.79 | 0.72 | 0.51-1.01 |  | 0.90 | 0.57-1.44 |  |
|  | Severe | 1.93 | 1.26-2.94 |  | 3.03 | 1.10-8.32 | 1.13 | 0.62-2.06 |  | 1.28 | 0.56-2.90 |  |
| Anxiety/depression | |  |  | 0.010† |  |  |  |  | 0.003† |  |  | 0.465 |
|  | No | 1 |  |  | 1 |  | 1 |  |  | 1 |  |  |
|  | Moderate | 1.39 | 1.13-1.72 |  | 0.60 | 0.33-1.07 | 0.63 | 0.46-0.86 |  | 1.07 | 0.65-1.77 |  |
|  | Extreme | 1.12 | 0.58-2.17 |  | 0.16 | 0.03-0.83 | 0.38 | 0.18-0.82 |  | 0.49 | 0.15-1.63 |  |
| Tympanic membrane | |  |  | <0.001† |  |  |  |  | 0.002† |  |  | <0.001† |
|  | Normal, both | 1 |  |  | 1 |  | 1 |  |  | 1 |  |  |
|  | Abnormal, unilateral | 2.29 | 1.79-2.90 |  | 1.75 | 0.98-3.14 | 0.76 | 0.56-1.04 |  | 0.65 | 0.41-1.03 |  |
|  | Abnormal, bilateral | 2.39 | 1.52-3.77 |  | 2.58 | 1.21-5.05 | 0.74 | 0.45-1.18 |  | 0.29 | 0.17-0.49 |  |
| Tinnitus | |  |  | <0.001† |  |  |  |  | <0.001† |  |  | 0.001† |
|  | No | 1 |  |  | 1 |  | 1 |  |  | 1 |  |  |
|  | Yes | 2.56 | 2.19-2.99 |  | 2.06 | 1.26-3.36 | 0.52 | 0.41-0.64 |  | 0.54 | 0.38-0.77 |  |

^*^Estimated rate, adjusted with weighted values

† Significance at P < 0.05
