# Supplementary material for: Discrepancy between self-assessed hearing status and measured audiometric evaluation
Source: PLoS One. 2017 Aug 8;12(8):e0182718. doi: 10.1371/journal.pone.0182718 (PMC5549722; doi:10.1371/journal.pone.0182718)
Supplement: S3 Table — (DOCX) [file pone.0182718.s003.docx]

**S3 Table** Differences in the general characteristics of participants underwent pure tone audiometry and those who did not

|  | |  | Pure tone audiometry | | P-value |
| --- | --- | --- | --- | --- | --- |
|  | |  | (-) | (+) |  |
| Number | | |  |  |  |
|  | N | | 7,095 | 20,097 |  |
|  | % | | 26.1 | 73.9 |  |
| Age (year) | | | 50.2 | 50.6 | 0.131 |
| Sex (%) | | |  |  | <0.001† |
|  | Male | | 27.2 | 72.8 |  |
|  | Female | | 25.3 | 74.75 |  |
| Education (%) | | |  |  | <0.001† |
|  | Low | | 23.1 | 76.9 |  |
|  | Middle | | 20.6 | 79.4 |  |
|  | High | | 18.9 | 81.1 |  |
| Stress level (%) | | |  |  | 0.020† |
|  | None | | 22.4 | 77.6 |  |
|  | Some | | 20.5 | 79.5 |  |
|  | Moderate | | 21.4 | 78.6 |  |
|  | Severe | | 22.8 | 77.2 |  |
| Anxiety/depression | | |  |  | <0.001† |
|  | No | | 21.1 | 78.9 |  |
|  | Moderate | | 19.8 | 80.2 |  |
|  | Extreme | | 31.1 | 68.9 |  |
| Tympanic membrane (%) | | |  |  | 0.137 |
|  | Normal, both | | 20.1 | 79.9 |  |
|  | Abnormal, unilateral | | 20.1 | 799 |  |
|  | Abnormal, bilateral | | 23.2 | 76.8 |  |
| Tinnitus (%) | | |  |  | 0.380 |
|  | No | | 20.4 | 79.6 |  |
|  | Yes | | 198.8 | 80.2 |  |

* ANOVA test, significance at P < 0.05

† Chi-square test, significance at P < 0.05
