# Supplementary material for: Discrepancy between self-assessed hearing status and measured audiometric evaluation
Source: PLoS One. 2017 Aug 8;12(8):e0182718. doi: 10.1371/journal.pone.0182718 (PMC5549722; doi:10.1371/journal.pone.0182718)
Supplement: S4 Table — (DOCX) [file pone.0182718.s004.docx]

**S4 Table** Hearing perception according to self-reported and audiometric hearing loss at the high frequency hearing threshold

| Self-reported hearing loss | Audiometric hearing threhold (high frequency threshold) | | |
| --- | --- | --- | --- |
|  | <25 dB | ≥25 dB, <40 dB | ≥40 dB |
| No difficulty | 11,654*  (94.1%) | 2,704‡  (83.8%) | 2,413‡  (59.9%) |
| Some difficulty | 681†  (5.5%) | 472*  (14.6%) | 1,171‡  (29.1%) |
| Much difficulty | 52†  (0.4%) | 50†  (9.1%) | 445*  (11.0%) |
| Total | 12,387  (100%) | 3,226  (100%) | 4,029  (100%) |

High frequency hearing threshold = (3000 Hz + 4000 Hz + 6000 Hz)/3

* Concordance of hearing impairment: No discomfort at <25 dB; some discomfort at ≥25 dB, <40 dB; much discomfort at ≥40 dB hearing threshold

†Overestimation of hearing loss: Some discomfort at <25 dB; much discomfort at both the <25 dB and ≥25 dB, <40 dB hearing thresholds

‡Underestimation of hearing loss: No discomfort at the ≥25 dB, <40 dB and ≥40 dB thresholds; some discomfort at the ≥40 dB hearing threshold
