# Supplementary material for: Discrepancy between self-assessed hearing status and measured audiometric evaluation
Source: PLoS One. 2017 Aug 8;12(8):e0182718. doi: 10.1371/journal.pone.0182718 (PMC5549722; doi:10.1371/journal.pone.0182718)
Supplement: S5 Table — (DOCX) [file pone.0182718.s005.docx]

**S5 Table** Multinomial logistic regression analysis with complex sampling (reference = concordance) in high frequency hearing threshold

| Related factors | |  | Overestimation | | Underestimation | |  |
| --- | --- | --- | --- | --- | --- | --- | --- |
|  | | %^*^ | AOR | 95% CI | AOR | 95% CI | P-value |
| Age (10 years) | |  | 1.17 | 1.07-1.29 | 2.80 | 2.66-2.93 | <0.001† |
| Sex | |  |  |  |  |  | <0.001† |
|  | Male | 49.7 | 1 |  | 1 |  |  |
|  | Female | 50.3 | 1.02 | 0.82-1.27 | 0.28 | 0.25-0.31 |  |
| Education | |  |  |  |  |  | <0.001† |
|  | Low | 27.4 | 1 |  | 1 |  |  |
|  | Middle | 45.1 | 0.85 | 0.66-1.09 | 0.71 | 0.62-0.80 |  |
|  | High | 27.6 | 0.83 | 0.58-1.20 | 0.50 | 0.41-0.60 |  |
| Occupation | |  |  |  |  |  | 0.010† |
|  | Specialized worker | 23.5 | 1 |  | 1 |  |  |
|  | Service worker | 20.1 | 1.87 | 1.35-2.61 | 1.13 | 0.95-1.35 |  |
|  | Manual worker | 20.8 | 1.50 | 1.05-2.13 | 1.13 | 0.95-1.34 |  |
|  | Unemployed | 35.6 | 1.61 | 1.22-2.13 | 0.98 | 0.84-1.15 |  |
| Stress | |  |  |  |  |  | 0.006† |
|  | None | 13.2 | 1 |  | 1 |  |  |
|  | Some | 58.6 | 1.23 | 0.88-1.73 | 1.05 | 0.91-1.21 |  |
|  | Moderate | 23.6 | 1.71 | 1.19-2.46 | 1.07 | 0.90-1.27 |  |
|  | Severe | 4.6 | 2.23 | 1.35-3.64 | 1.06 | 0.82-1.38 |  |
| Anxiety/Depression | |  |  |  |  |  | 0.046† |
|  | None | 89.8 | 1 |  | 1 |  |  |
|  | Moderate | 9.6 | 1.32 | 1.01-1.72 | 0.89 | 0.75-1.06 |  |
|  | Extreme | 0.6 | 0.78 | 0.34-1.83 | 1.31 | 0.74-2.33 |  |
| Tympanic membrane | |  |  |  |  |  | <0.001† |
|  | Normal, both | 91.2 | 1 |  | 1 |  |  |
|  | Abnormal, unilateral | 6.4 | 2.09 | 1.50-2.91 | 1.04 | 0.85-1.26 |  |
|  | Abnormal, bilateral | 2.3 | 2.64 | 1.61-4.31 | 0.93 | 0.68-1.27 |  |
| Hearing aid use | |  |  |  |  |  | <0.001† |
|  | No | 99.4 | 1 |  | 1 |  |  |
|  | Yes | 0.6 | 0.17 | 0.06-0.45 | 0.68 | 0.04-0.13 |  |
| Tinnitus | |  |  |  |  |  | <0.001† |
|  | No | 79.2 | 1 |  | 1 |  |  |
|  | Yes | 20.8 | 2.60 | 2.14-3.17 | 1.45 | 1.27-1.65 |  |

^*^ Estimated rate, adjusted with weight values

† Significance at P < 0.05

High-frequency hearing threshold = (3000 Hz + 4000 Hz + 6000 Hz)/3
